# Ancestral Stress Alters Lifetime Mental Health Trajectories and Cortical Neuromorphology via Epigenetic Regulation

**DOI:** 10.1038/s41598-019-42691-z

**Published:** 2019-04-23

**Authors:** Mirela Ambeskovic, Olena Babenko, Yaroslav Ilnytskyy, Igor Kovalchuk, Bryan Kolb, Gerlinde A. S. Metz

**Affiliations:** 1 Canadian Centre for Behavioural Neuroscience, Department of Neuroscience, University of Lethbridge, 4401 University Drive, Lethbridge, Alberta T1K 3M4 Canada; 2 Department of Biological Sciences, University of Lethbridge, Lethbridge, Alberta T1K 3M4 Canada

**Keywords:** Neuronal development, Anxiety, Prefrontal cortex, Epigenetics and behaviour

## Abstract

Experiences during early development are powerful determinants of lifetime mental health. Here we investigated if ancestral stress regulates the brain’s epigenetic memory to alter neuromorphology and emotionality in the remote F4 progeny. Pregnant female rat dams of the parental F0 generation were exposed to stress on gestational days 12–18. To generate a transgenerational stress lineage, their pregnant daughters (F1), grand-daughters (F2) and great-grand-daughters (F3) remained undisturbed. To generate a multigenerational stress lineage, pregnant dams of each generation (F1–F3) were stressed. A lineage of non-stress controls (F0–F3) was also produced. Multigenerational stress exceeded the impact of transgenerational stress by increasing anxiety-like behaviours and stress response in young and middle-aged F4 males but not females. Functional changes were accompanied by reduced spine density in the male medial prefrontal cortex with opposite effects in the orbital frontal cortex. Ancestral stress regulated cortical miR-221 and miR-26 expression and their target genes, thus downregulating *ntrk2* and *map1a* genes in males while downregulating *crh* and upregulating *map1a* genes in females. These miRNA-dependent pathways are candidates for developmental programming of lifetime mental health. Thus, multigenerational stress in particular determines sexually dimorphic predisposition to stress vulnerability and generates a phenotype resembling symptoms of post-traumatic stress disorder.

## Introduction

The prevalence of mental health disorders worldwide has surged to 36.7% in the last decade^1^. Evidence suggests that the exposure to adverse environments during early development plays a role in programming mental illness, such as attention deficit hyperactive disorder (ADHD), anxiety, depression, and vulnerability to post-traumatic stress disorder (PTSD)^2^. The developmental origins of health and disease (DOHaD) theory posits that the exposure to adverse environments during the fetal period and early childhood can program the vulnerability to disease in later life^3^. Accordingly, numerous studies have shown that prenatal stress, undernutrition, and perinatal inflammation may alter the stress response and behavioural phenotype leading to increased risk of psychiatric disorders later in life^2,4–6^.

Prenatal exposure to stress in particular alters fetal brain development which manifest in behavioural alterations associated with mental health disorders later in life. For example, human studies have reported increased incidence of anxiety and depression in children born to mothers who experienced distress, such as war, family violence, natural disaster or death of a close relative, during pregnancy^2,7,8^. Similarly, animal models of prenatal stress have shown increased latency to play^9^, and anxiety-like behaviours^10^ in offspring. Stress programming is likely associated with altered neuronal plasticity^11,12^. Reports demonstrated alterations in spine density and dendritic branching in both medial prefrontal cortex (mPFC) and orbital frontal cortex (OFC), two frontal regions associated with emotional behaviours^5,12^ with significant sexual dimorphisms^13,14^.

Human and animal studies support the notion of inter-generational programming by prenatal stress. Prenatal undernutrition has life-long impact on health and disease incidence of children and grand-children^15,16^. Moreover, ancestral prenatal exposure to elevated steroid hormones and toxins alters behaviour and disease incidence in unexposed grand offspring^17,18^. Ancestral prenatal stress across generations manifests itself in reduced maternal activity pre-partum^19^, impaired offspring sensorimotor development that becomes recognizable as early as P7^20^ and altered fine motor function in adulthood^20,21^. Functional impairments were accompanied by distinct sex-specific metabolic profiles with consistent upregulation of hippurate and downregulation of tyrosine, threonine, and histamine^22^ and altered neuronal epigenetic regulators such as miR-200 and miR-181 expression^20,23^. Ancestral epigenetic programming has been ascribed an important role in disease risk^17,24^. Thus, we hypothesized that through epigenetic regulation of cortical microRNA (miRNA) expression, a stressful environment may result in a transgenerational and multigenerational neuromorphological, endocrine and emotional phenotype. This mechanism may explain why the mental illness may occur in the absence of genetic risk factors, such as indicated for the *ntrk2* single nucleotide polymorphisms in PTSD^25,26^.

The present study examined in rats if ancestral stress exposure in a single generation vs. multiple generations produces behavioural and endocrine phenotypes that (1) persist to the remote F4 generation, and (2) affect the offspring at different ages. The experiment was designed to investigate sexually dimorphic patterns in stress reactivity and neuroplastic adaptations in the medial prefrontal cortex (mPFC) and orbital frontal cortex (OFC) during early and late adulthood. The findings show that recurrent prenatal stress exposure over four generations (multigenerational stress) exceeded the effects of single generation stress (transgenerational) in induced anxiety-like behaviour. The present findings suggest miRNA dependent pathways may play role in sex-specific transgenerational programming of lifelong stress vulnerability and resiliency.

## Results

### Transgenerational and multigenerational stress increased levels of anxiety-like behaviours in male but not female F1-F4 generation offspring at P90

**Figure 1:**
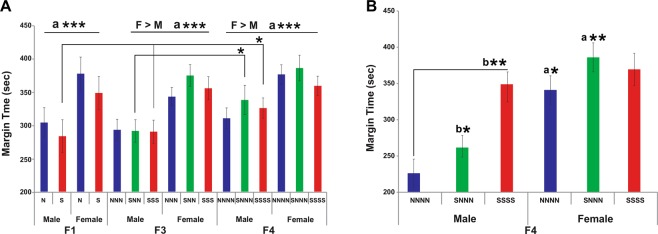
Effects of prenatal stress across generations on anxiety-like behaviour. Anxiety-like behaviour was indicated by the amount of time a rat spent in the margins of an open field task. (**A**) Both trans- and multigenerational stress increased levels of anxiety-like behaviours in F3 and F4 generation male but not female rats at postnatal day (P) 90. (**B**) Multigenerational stress cumulatively exacerbated anxiety-like behaviours, as F4-SSSS males spent more time in margins than SNNN (transgenerational) or NNNN (non-stressed) rats at P180. Asterisks indicate significances: *P < 0.05, **P < 0.01, ***P < 0.001. All data presented as mean ± SEM. “a” indicates sex effect; “b” indicates stress effect.

Ancestral prenatal stress across generations cumulatively induced anxiety-like behaviours in F3 and F4 generation male offspring that were subjected to transgenerational stress (SNNN) or multiple repeated stress or multigenerational stress (SSSS; P < 0.05). Females showed overall higher level of anxiety-like behaviours than males across all generations, however no significant effects of ancestral stress were observed in females. A three-way ANOVA with Sex, Generation, and Stress as factors revealed a main effect of all three factors individually, but no interactions. The time spent in margins of the open field revealed a significant main effect of Sex (F(1, 261) = 39.87, P < 0.001, Fig. 1A), as females overall spent more time in margins than males when F1-F4 generation offspring were combined. Moreover, when each generation was observed separately, significant sex differences were found in F1 and F3 generations (P < 0.001), while smaller, but significant, differences were found between male and female offspring in the F4 generation (P < 0.05; Fig. 1A). These data indicate that multiple exposures to stress either through transgenerational or multigenerational transmission has fewer effects on anxiety-like behaviours in females than males and may demasculinize male behaviour. For instance, multigenerational stress increased the levels of anxiety in males to nearly comparable levels observed in females, as indicated by increased time spend in margins in F4 generation males. Increased margin time at the expense of centre time generally indicates higher anxiety-like behaviour.

Time spent in margins of the open field revealed a significant main effect of Generation (F(2, 260) = 3.15, P < 0.05; Fig. 1A), as the time in margins increased in later generations. For example, the offspring belonging to F1 and F3 generations spent significantly less time (P < 0.05) in margins than the F4 generation rats. Moreover, time spent in margins revealed a main effect of Stress ((F2, 260) = 3.15, P < 0.05; Fig. 1A), as overall stressed male and female rats spent significantly more time in margins of an open field than controls. Notably, when the effects of stress were observed within each generation individually, the stress had most profound effect on the time spent in margins in the F4 generation as suggested above. Stress has slightly but nonsignificant decreased time spent in margins in both males (P > 0.05) and females (P > 0.05) when compared to non-stressed rats. Stress-induced a non-significant decrease in the time spent in margins in the F3 multigenerational and transgenerational males (P > 0.05) and an increase in females (P > 0.05; Fig. 1A) in comparison to non-stressed rats. Although not significant, the largest effect of stress was observed in the F4 generation, where stress increased the time spent in margins in both males (P > 0.05) and females (P > 0.05). Since the largest effect was observed in F4 generation offspring at P 90, these animals were aged and tested again to determine if differences persist.

### Multigenerational but not transgenerational stress cumulatively increased levels of anxiety-like behaviours in male F4 offspring at P180

Ancestral exposure to stress across four generations significantly increased time spent in margins in multigenerationally stressed (SSSS) but not transgenerationally stressed (SNNN) male rats or females. Interestingly, multigenerationally stressed males show similar behaviour profiles as compared to females.

A two-way ANOVA revealed a main effect of Stress and Sex, and their interaction. The time spent in margins of the open field revealed a significant main effect of Sex (F(1, 53) = 17.9, P < 0.000; Fig. 1B), as female rats overall spent more time in the margins than male offspring. Moreover, stress had an almost significant (F(2, 52) = 2.94, P = 0.062; Fig. 1B) effect on the time spent in margins. A pairwise comparison showed that both transgenerationally (F4-SNNN, P < 0.05) and multigenerationally (F4-SSSS, P < 0.05) stressed rats spent more time in margins than non-stressed ones (F4-NNNN). However, a Tukey posthoc test comparing the effect of stress within their respective sex revealed a significant effect in multigenerationally stressed (F4-SSSS) males only. When compared to non-stressed males (F4-NNNN), transgenerationally stressed (F4-SNNN, P = 0.95) males did not spend more time in the margins, while multigenerationally stressed ones did (F4-SSSS, P > 0.05; Fig. 1B). Neither transgenerationally (F4-SNNN, P = 0.37) nor multigenerationally (F4-SSSS, P = 1) stressed females spent significantly more time in margins than non-stressed (F4-NNNN) rats. Time spent in margins also showed a significant interaction Sex x Stress (F(2, 52) = 4.0, P < 0.05; Fig. 1B), as transgenerational stress prolonged the time spent in margins in females (F4-SNNN), while decreasing it in males (F4SNNN) when compared to multigenerationally stressed rats.

### Multigenerational stress increased anxiety-like behaviours in F4 generation males at P180 in the elevated plus maze

**Figure 2:**
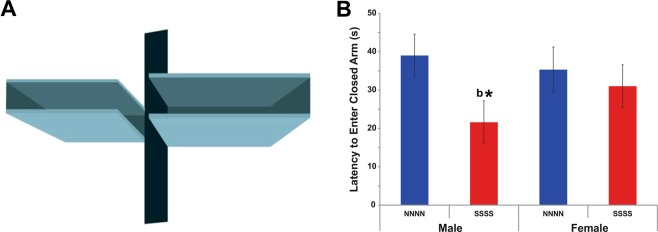
Effects of multigenerational prenatal stress on anxiety-like behaviours in the elevated plus maze (EPM) at P180. Anxiety-like behaviour was indicated by the latency to enter or escape into a closed arm. (**A**) A representative photograph of a rat escaping into a closed arm of the EPM. (**B**) Multigenerational stress decreased latency to enter closed arm in F4-SSSS males in comparison to non-stressed males. Asterisks indicate significances: *P < 0.05. All data are presented as mean ± SEM. “b” indicates stress effect.

Because cumulative effects of multigenerational stress here showed the largest impact at P90, additional analyses on aging animals at P180 were performed for the F4-SSSS generation to further discover sexual dimorphisms. Recurrent stress across multiple generations in males decreased their latency to enter closed arms, while it had no effect on females. Two-way ANOVA revealed a main effect of Stress as overall stressed male and female offspring were significantly (F(1, 35) = 3.9, P = 0.05; Fig. 2) faster to enter closed arms than non-stressed rats.

Moreover, latencies in stressed males were significantly shorter than non-stressed males (t(16) = 2.11, P < 0.05; Fig. 2), stressed (t(16) = 3.88, P < 0.001) and non-stressed (t(16) = 3.16, P < 0.01; Fig. 2) females. Sex X Stress interaction showed a trend (F(1, 35) = 3.5, P = 0.069; Fig. 2) with stress moderately increasing latencies in females but decreasing it in males.

### Multigenerational stress blunted basal circulating corticosterone levels in F4 generation males only

**Figure 3:**
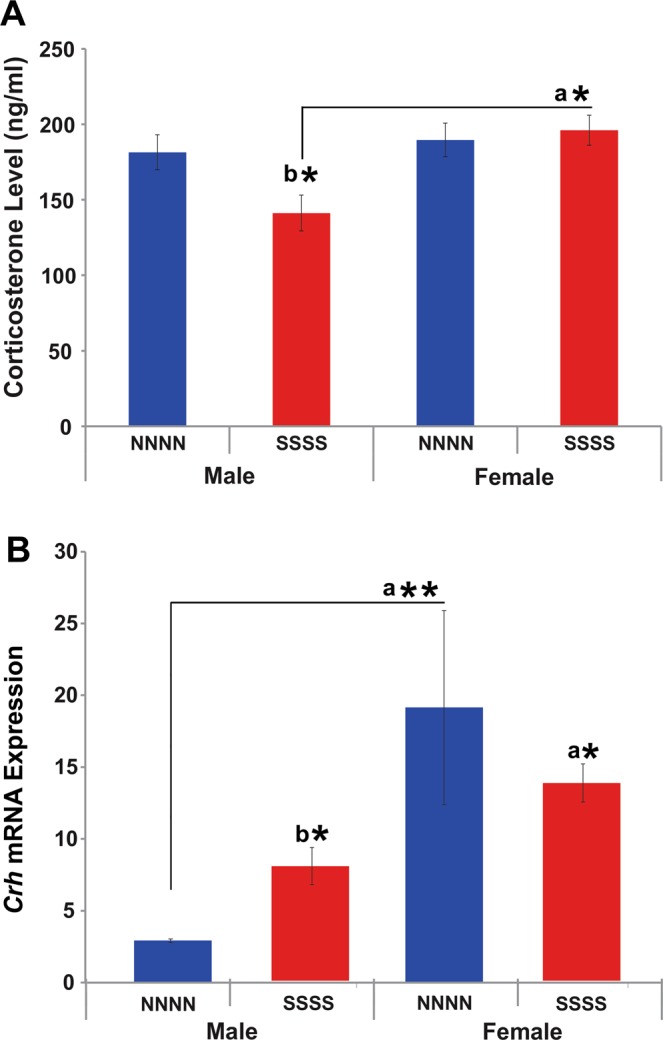
Effects of multigenerational stress on stress response systems. (**A**) Multigenerational stress reduced circulating corticosterone levels in males, while no changes were observed in female rats. (**B**) Deep sequencing of frontal cortex revealed that multigenerational stress upregulated *Crh* mRNA expression in males, with slight but non-significant downregulation in female rats. Asterisks indicate significances: *P < 0.05, **P < 0.01. All data are presented as mean ± SEM. “a” indicates sex effect; “b” indicates stress effect.

A two-way ANOVA with Sex and Stress as factors revealed a main effect of Sex. Overall significantly higher levels of circulating corticosterone were found in female rats (F(1, 35) = 5.34, P < 0.05; Fig. 3A). Moreover, a trend of interaction between Sex and Group (F(1, 35) = 3.77, P = 0.061; Fig. 3A) indicated moderately higher corticosterone levels in female and reduced levels in males rats. An independent sample-test revealed increased corticosterone levels in stressed males t(16) = 2.61, P < 0.05) but not non-stressed (t(16) = 2.61, P < 0.05; Fig. 3A) male controls, non-stressed females (t(16) = 2.86, P < 0.05) or stressed female (t(16) = −2.66, P < 0.05).

### Multigenerational stress altered neuromorphology of orbital frontal cortex (OFC) and medial prefrontal cortex (mPFC) in F4 generation males and females

Repeated exposure to stress across multiple generations induced opposite effects in medial prefrontal cortex (mPFC or Cg3) and orbitofrontal cortex (OFC or AID) neuromorphology. A three-way ANOVA revealed a main effect of Sex (F(2, 46) = 25.0, P < 0.001), Stress (F(2, 46) = 4.2, P < 0.01), and nearly significant effect of Hemisphere (F(2, 46) = 2.2, P = 0.066) for the Cg3. Spine density in males was higher than in females (F(1, 47) = 52.9, P < 0.001), while dendritic length and branching were unaffected by sex. Stress decreased branch order and spine density.

Dendritic branching in the basilar field revealed a significant main effect of Hemisphere (F(1, 47) = 4.7, P < 0.05) as the right hemisphere had more elaborate branching than the left. Moreover, multigenerational stress induced a non-significant increase in dendritic branching (F(1, 47) = 3.4, P = 0.07) in the apical field of both hemispheres and sexes in comparison to non- stressed controls.

**Figure 4:**
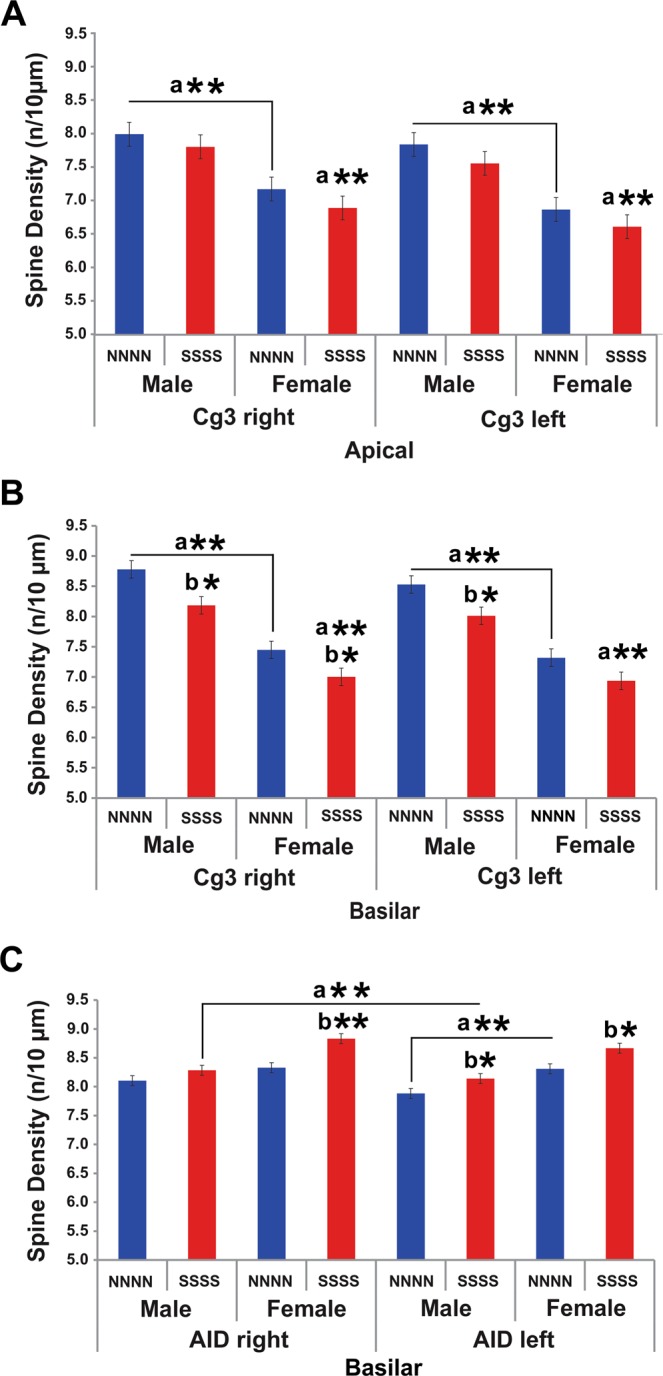
Dendritic spine organization of the medial prefrontal cortex (Cg3) and orbital frontal cortex (AID) in response to multigenerational stress. Multigenerational stress induced sexually dimorphic effects in Cg3 and AID dendritic spine density. (**A**) Cg3 spine density in the apical field was higher in non-stressed and stressed males compared to females. (**B**) Multigenerational stress decreased the number of spines in the Cg3 basilar field in male and female rats. Males had higher spine density than stressed (SSSS) and non-stressed (NNNN) females. (**C**) Mutigenerational stress increased the number of spines in the AID in males and females. Cg3 dendritic spine density in females were higher than in males. Multigenerationally stressed females had most dendritic spines in the right hemisphere of the AID. Asterisks indicate significances: *P < 0.05, **P < 0.01. All data are presented as mean ± SEM. “a” indicates sex effect; “b” indicates stress effect.

Dendritic length in the apical field exhibited a significant Sex x Stress interaction (F(1, 47) = 6.3, P < 0.05), as stress diminished dendritic length in the left hemisphere of males but exacerbated it in females. For spine density, apical dendrites exhibited a significant effect of Hemisphere (F(1, 47) = 3.85, P = 0.057; Fig. 4A,B), as the right hemisphere had longer dendrites than the left. A significant effect of Sex was found in both apical (F(1, 47) = 52.9, P < 0.001) and basilar (F(1, 47) = 138.6, P < 0.001; Fig. 4A,B) fields, as males had larger spine density than females. Importantly, multigenerational stress significantly reduced basilar spine density in both right (Male: t(10) = 2.14, P < 0.05; Female: t(10) = 3.17, P < 0.05; Fig. 4A,B; Table 1) and left (Male: t(10) = 2.73, P < 0.05; Female: t(10) = 2.25, P < 0.05; Table 1) hemispheres of F4 males and females when compared to non-stressed (NNNN) rats.

**Table 1:** Summary of dendritic branching, length and spine density results in the Cg3 region of the mPFC and the AID region of the OFC. The arrows represent the direction of effects in response to stress (relative to controls).

	MALE		FEMALE	
	Right Hemisphere	Left Hemisphere	Right Hemisphere	Left Hemisphere
	SSSS	SSSS	SSSS	SSSS
**Dendritic Branching**				
Cg3 Apical	↑	↓	↑	↑
Cg3 Basilar	↓	↓	↓	↑
AID Basilar	↓	↓	↑	↓
**Dendritic Length**				
Cg3 Apical	↓	↓*	↑	↑
Cg3 Basilar	↓	↓	↓	↑
AID Basilar	↓	↓	↑	↑
**Dendritic Spine Density**				
Cg3 Apical	↓	↓	↓	↓
Cg3 Basilar	↓*	↓*	↓*	↓
AID Basilar	↑	↑	↑**	↑

Asterisk indicate significant differences, *p < 0.05, **p < 0.01.

For the orbital frontal cortex (AID), a three-way ANOVA revealed main effects of Sex (F(2, 46) = 18.3, P < 0.001; Fig. 4C) and Stress (F(2, 46) = 9.21, P < 0.001; Fig. 4C). Females exhibited predominantly longer dendrites with more spines than males. Stress overall slightly decreased complexity and length of dendrites in AID, while increasing the number of spines. Both right and left hemispheres had similar dendritic branching and length; however, the right hemisphere of both males and females had more spine density. Specifically, spine density in AID exhibited a main effect of Hemisphere (F(1, 47) = 5.1, P < 0.05; Fig. 4C), Sex (F(1, 50) = 50.1, P < 0.001; Fig. 4C) and Stress (F(1, 47) = 28.24, P < 0.001; Fig. 4C) but no significant interactions. Importantly, multigenerational stress increased spine density in both right (Male: t(10) = −2.18, P < 0.05; Female: t(10) = −3.86, P < 0.05) and left (Male: t(10) = −2.32, P < 0.05; Female: t(10) = −2.32, P < 0.05; Fig. 4C, Table 1) AID hemispheres of both F4 generation males and females.

### Multigenerational stress altered epigenetic regulation through miR-221 and miR-26 and its target genes in prefrontal cortex tissue in F4 generation males

**Figure 5:**
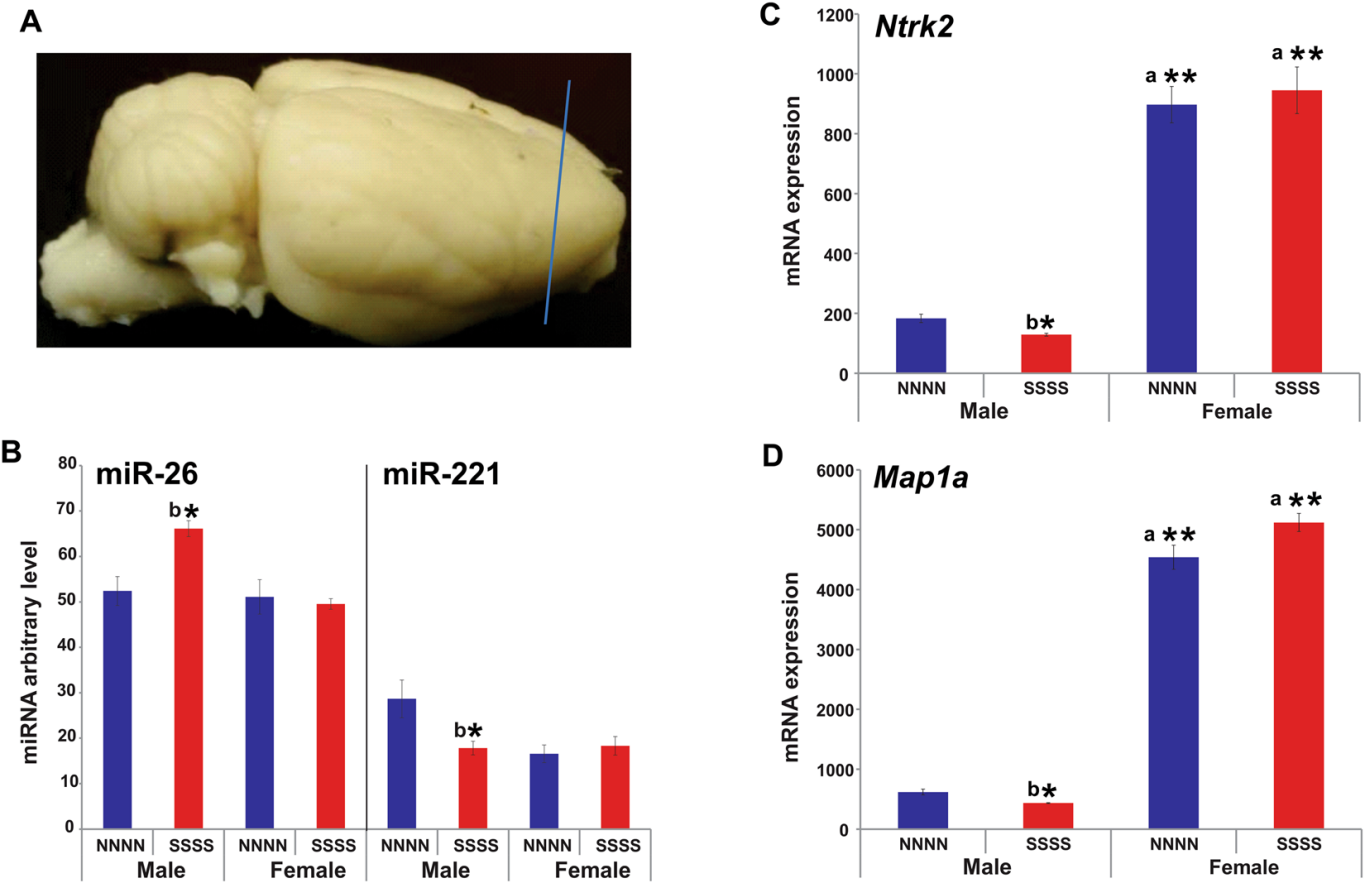
MiRNA and mRNA expression in the frontal cortex. Multigenerational stress altered epigenetic regulation of miR-221 and miR-26 and mRNA (*ntrk2*, *map1a*) in a sex-specific manner. (**A**) Location of frontal cortex tissue sample used for deep sequencing. (**B**). Multigenerational stress downregulated miR-221expression and upregulated miR-26 expression in males. (**C**) Multigenerational stress significantly decreased the *ntrk2* mRNA expression in males, while a slight upregulation was observed in female rats. Females showed four times higher expression of *Ntrk2* than males. (**D**) Multigenerational stress downregulated *map1a* expression in males. Asterisks indicate significances: *P < 0.05, **P < 0.01. All data are presented as mean ± SEM. “a” indicates sex effect; “b” indicates stress effect.

Deep sequencing revealed that two miRNAs of interest linked to anxiety-like behaviours through regulation of neuronal proliferation and plasticity were differentially expressed in the frontal cortex in response to stress. Stress downregulated miR-221 and upregulated miR-26 expression in multigenerational stress males (SSSS; FDR P < 0.05 adjusted using the Benjamin and Hochberg correction; Fig. 5B). Expression of these miRNAs was not significantly affected in females. Thus, miR-26 and miR-221 expression showed sexually dimorphic effects. Moreover, multigenerational stress upregulated gene expression for corticotrophin releasing hormone *(crh;* Fig. 3B*)*, and downregulated neurotrophic receptor tyrosine kinase 2 (*ntrk2;* Fig. 5C*)*, and microtubule-associated protein 1a *(map1a;* Fig. 5D) gene expression in male (SSSS) offspring. Interestingly, miR-26 is an up-stream regulator of *ntrk2* and *map1a* expression^27^.

## Discussion

**Figure 6:**
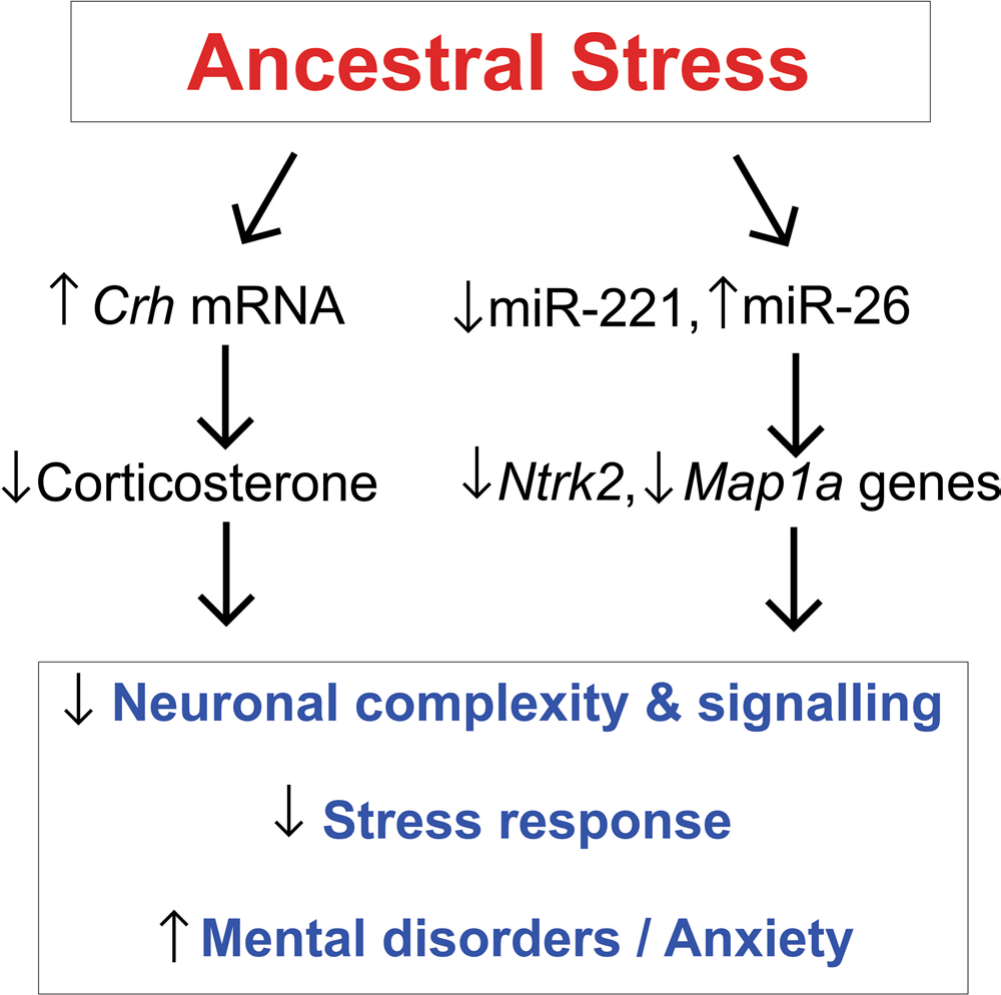
Diagram illustrating potential epigenetic mechanisms by which ancestral stress may regulate neuromorphology and mental health. In pathway #1 ancestral stress alters expression of miR-221 and miR-26. Upregulated mir-26 results in downregulated *ntrk2* and *map1a* expression which ultimately reduces neuronal complexity and adaptive stress response. In pathway #2 ancestral stress alters *crh* mRNA expression in frontal cortex that leads to HPA axis impairment with blunted corticosterone levels, as found in PTSD. This ultimately alters neuronal morphology and stress system as hallmarks of mental health.

The present findings support the notion that ancestral stress may program mental health outcomes via epigenetic regulation of neuroendocrine activity and neuronal spine density in males. Here we provide four main supportive arguments. First, both transgenerational and multigenerational ancestral stressors were characterized by an altered affective state in early adulthood. However, multigenerational stress exceeded the effects of transgenerational stress by increasing anxiety-like behaviours in older adult male but not female rats. Second, multigenerational stress altered stress responsiveness, and reduced corticosterone levels in males only. Third, multigenerational stress-related functional and physiological changes were accompanied by changes to dendritic branching and spine density of prefrontal cortex (mPFC and AID). Multigenerational stress reduced spine density in the mPFC but increased it in the AID in both male and female offspring. Fourth, deep sequencing revealed that stress altered miR-221, miR-26 and mRNA expression of some of their target genes (*crh, ntrk2, and map1a*) in frontal cortices. The latter findings indicate that epigenetic regulation may be causally related to behavioural, neuroendocrine and morphological pathophysiologies of mental health (Fig. 6).

The present observations corroborate the idea that adverse early life experiences, such as prenatal stress, are major determinants of brain development and mental health. Human studies have demonstrated that children exposed to prenatal stress are more vulnerable to emotional disturbances in adolescence and adulthood than non-exposed children^28^. Similarly, rodent studies revealed that exposure to prenatal or chronic postnatal stress raises levels of anxiety-like behaviours, such as spending less time than controls in the open arms of the EPM^29^ and enhanced freezing^30^.

Recent experimental and clinical studies have demonstrated that exposure to adverse environments early in life may propagate across generations to alter emotionality in the unexposed offspring^22,31,32^. Here we show that exposure of mothers, grandmothers and even great-grandmothers to stress during pregnancy is transmitted to their indirectly (transgenerational) or directly (multigenerational) exposed offspring in terms of phenotype traits.

Specifically, both trans- and multigenerational stress increased anxiety-like behaviours in F3 and F4 generation male offspring only. Similarly, Franklin *et al*.^33^, showed that exposing great-grandfathers to unpredictable postnatal maternal separation increased emotionality in F3 transgenerationally stressed male and F2 female mice. Moreover, Dias and Ressler^34^, reported that F1 and F2 generation offspring whose fathers and grandfathers were subjected to odor fear conditioning during adult life exhibited increased sensitivity to the same odor, even if they never experienced it themselves. The present findings add and expand on these observations by suggesting that both transgenerational and multigenerational ancestral stress can affect the affective state of adult offspring in a sexually dimorphic manner. In addition, recurrent prenatal stress in the multigenerationally stress lineage seems to exacerbate the effects of transgenerational stress. As emotionality is a prominent behavioural trait with evolutionary importance, ancestral transmission of stress to future progeny may serve as a way to build resilience^35^, and improve fitness and survival via epigenetic memory.

Our results indicate that each additional generation of gestational stress via maternal lineage incrementally exacerbated anxiety-like behaviours, with the F4 multigenerationally stressed male offspring experiencing the largest change. Our previous animal cohorts support vulnerability of multigenerationally stressed lineages to anxiety-like states^22^ and hyperactivity^36^ in adult F4 males. The compounding effects of prenatal stress across generations seem to be very profound and sex-specific. For example, our earlier study reported impairments in skilled reaching movements in male and improvements in female multigenerationally stressed F4 rats^21^. Multigenerationally stressed F4 females commonly display higher stress resiliency unless faced by another challenge^35^ suggesting a dysfunctional stress response system as the core issue^20,23,35^. It is well established that ancestral stress elevates hypothalamic-pituitary adrenal axis activity due to impaired negative feedback loop^37^ permanently altering brain morphology and behaviour^21,24,33,38^.

Here we report changes in the anxiety-like behavior and the stress responsiveness via corticosterone levels and *Crh* expression observed in the multigenerationally stressed males were accompanied by the dendritic reorganization of functionally meaningful areas, the medial prefrontal cortex (mPFC) and the orbital frontal cortex (OFC). Multigenerational stress increased dendritic spine density in both right and left OFC hemisphere in female and only in left OFC in male offspring. On contrary, stress-induced decrease in dendritic spine density of the basilar field mPFC in both sexes. Earlier findings showed a decrease in dendritic spine density in the parietal cortex of multigenerationally stressed offspring^21^. Accordingly, prenatal and chronic stress can have lasting effects on the neuromorphology of the prefrontal cortex^12,39^. Mychasiuk *et al*.^40^ found that prenatal stress decreased dendritic length in the OFC in juvenile male and female rats but had no effects on the mPFC. Moreover, prenatal stress increased spine number in mPFC and decreased it in OFC in males, while the opposite was observed in females^14^. Prenatal stress resulted in decrease in cortical spine density in the OFC in both males and females^13^. These observations indicate that stress particularly programs basilar dendritic spine density which may then reflect in the behavioural phenotype^39^ which may be due to vulnerability of thin spines to stress^41^. Decreased spine density might be related to multigenerational stress altering the functional signaling among cortical regions such as mPFC^42^ and reflect homeostatic reaction of the dendritic arbour to stress during early stages of connectivity^43^.

The multigenerational stress across four generations altered stress responsiveness especially in males. Specifically, stress diminished circulating corticosterone levels and upregulated expression of corticotrophin-releasing hormone *(Chr)* gene in the frontal cortex of males, while corticosterone levels and *Chr* expression were not affected in females. Accordingly, McCreary and colleagues^23^ demonstrated that multigenerational stress incrementally elevated corticosterone levels in young adult female (F1-F3) offspring. In a sheep study where pregnant mothers were injected with synthetic glucocorticoids during pregnancy, Long and colleagues^38^ reported increased levels of cortisol and adrenocorticotropic hormone in the F1 and F2 generation female offspring^38^. Similarly, a synthetic glucocorticoid injection to pregnant guinea pigs altered concentrations of cortisol, corticotropic releasing hormone (CRH) and hippocampal glucocorticoid receptor expression in F2 male and female offspring^18^. These changes may functionally explain phenotypic differences across ages, sexes and generations, with males generally being more susceptible^5,21,23,30^.

Epigenetic regulation of the *Crh* gene may serve as a mechanism of ancestral programming of HPA axis activity. CRH plays a prominent role in HPA axis regulation and downstream adrenal corticotropin-releasing hormone (ACTH)^44–46^. *Crh* gene expression in both the frontal cortex and amygdala can promote anxiety-like behaviours^47^. In turn, microinjecting a CRH antagonist^48^ or knocking out the hypothalamic *Crh* gene^49^ suppress anxiety-like behaviour.

This phenotype may be mediated by miRNAs as upstream epigenetic regulators of stress response. Here we showed here that multigenerational stress downregulated expression of miR-221 and upregulated expression of miR-26 in males, while no significant changes were observed in females. Mir-221 is found in distal axons where it targets mRNAs involved in neuronal communication and differentiation, neurite outgrowth and neurogenesis pathways^50,51^. Upregulation of miR-221 facilitates formation of neurite networks and synapses^50,52^. Similarly, miR-26 is enriched within neuronal dendrites and spines in the forebrain where it regulates synaptic plasticity associated with long-term potentiation (LTP)^51–53^. Reduced miR-26 expression is required for LTP maintenance, spine formation and enlargement^52^. Consistent with the present findings studies have also found that stress downregulates miR-221^54^ and upregulates miR-26^55,56^. Moreover, adverse experience such as exposure to cocaine also downregulates miR-221 expression in striatum but this change was reversed by enriched environment^57^. Importantly, miR-26 targets genes such as neurotrophic receptor tyrosine kinase 2 (*ntrk2*), and microtubule associated protein 1a (*map1a*), further regulating neuronal growth, maintenance and communication^27,50,51,53^.

Multigenerational stress also downregulated the expression of neurotropic tyrosine kinase receptor 2 (*ntrk2*), and microtubule-associated protein 1a (*map1a*) in male offspring only. The Ntrk2 or TrkB receptor for BDNF is critical in mediating activity-dependent synaptic plasticity and LTP^53,56^. Downregulation of *ntrk2* expression may decrease BDNF activity thus diminishing chances to generate LTP and synaptic plasticity. Similarly, downregulation of *map1a* gene would result in reduced LTP, as it is involved in functional maintenance and LTP related plasticity in mature neurons^57^. Moreover, reduced expression of *map1a* may result in remodeling of dendritic arbours and reduced density of active spines and synaptic surface density^54,57,58^. Thus, altered regulation of the target genes *ntrk2, map1a* and miR-221 and miR-26 may explain the sexually dimorphic neuromorphological findings and functional consequences on behaviour. Moreover, change in *ntrk2* expression is believed to be involved in synaptic plasticity and mental processes underlying psychopathology. Indeed, multiple studies have demonstrated association between *ntrk2* and psychiatric disorders^59–61^. Previous findings have linked abnormal *Ntrk2* expression to higher PTSD risk and suggested that it serves as one of the predictive biomarkers for risk for developing PTSD following trauma^22^ or as predictor of therapeutic response^25^. The present findings in rats show certain parallels to human PTSD, including abnormal HPA axis regulation and blunted stress response, and suggest that in the absence of DNA sequence variations epigenetic mechanisms may contribute to PTSD risk.

Multigenerational stress across four generations affected anxiety-like behaviours, neuronal re-organization and HPA axis activity likely via epigenetic regulation in a sex-specific manner. Sex specific effects were reported previously by showing region- and sex-specific changes in dendritic organization and spine density^11,14,21^, including decreased length and spine density in the mPFC^13^. Prenatal stress can induce anxiety-like behaviours especially in male rats in association with HPA axis dysfunction^5,44^. Mechanisms for these sexual dimorphisms may include interaction between Crh with estrogen and its receptors (ER alpha and ER beta)^62^. Because estrogen receptors are found in various brain regions, the *crh* gene has been shown to be important target of these steroids and potential mediator of sexually dimorphic stress response^62^. Since estradiol regulates *crh* expression via ER alpha and ER beta pathways transgenerational stress can epigenetically promote dysmasculinization and upregulation of ER alpha and ER beta receptors in male mice^63^. Moreover, estrogen receptors regulate both miR-221^64^ and miR-26 expression^65^. For example, ER-alpha can directly bind to the promotor region of miR-221 to suppress its expression^64^. Thus, transgenerational programming of steroid hormone actions may provide further insights into sex-specific emotional resilience or sensitivity to stress.

In conclusion the present findings are the first to show that the prenatal stress phenotype is epigenetically transmitted and accumulated across generations to resemble features of PTSD as indicated by altered *ntrk2* expression^26^ abnormal HPA axis regulation and blunted stress response^4^, especially in males. Downstream regulation of *ntrk2* and *map1a* by miR-26 resulted in impaired neuronal connectivity and increased incidence of symptoms associated with mental illness. We propose that epigenetic mechanisms such as miR-221 and miR-26 may be regulating the male phenotype and anxiety-like pathology and serve as potential biomarkers of stress vulnerability and mental illness. Furthermore, the data suggest that epigenetic mechanisms, in the absence of DNA sequence variations, contribute to PTSD risk and other adverse health outcomes.

## Methods

### Animals

Five generations of Long-Evans hooded rats were bred and raised at the Canadian Centre for Behavioural Neuroscience vivarium under carefully controlled conditions. For the present study 280 adult rats were housed in groups (males in pairs, females three per cage) under a 12:12 h light/dark cycle with light starting at 07:30 h and the room temperature set at 22 °C. All rats were tested on postnatal day (P) 90 (early adulthood) and left undisturbed except for weekly weighing and cage changes until P180. A subset of animals was tested in mid adulthood at six months of age as indicated. All procedures were approved by the University of Lethbridge Animal Care Committee in compliance with the guidelines by the Canadian Council on Animal Care.

### Experimental Design

**Figure 7:**
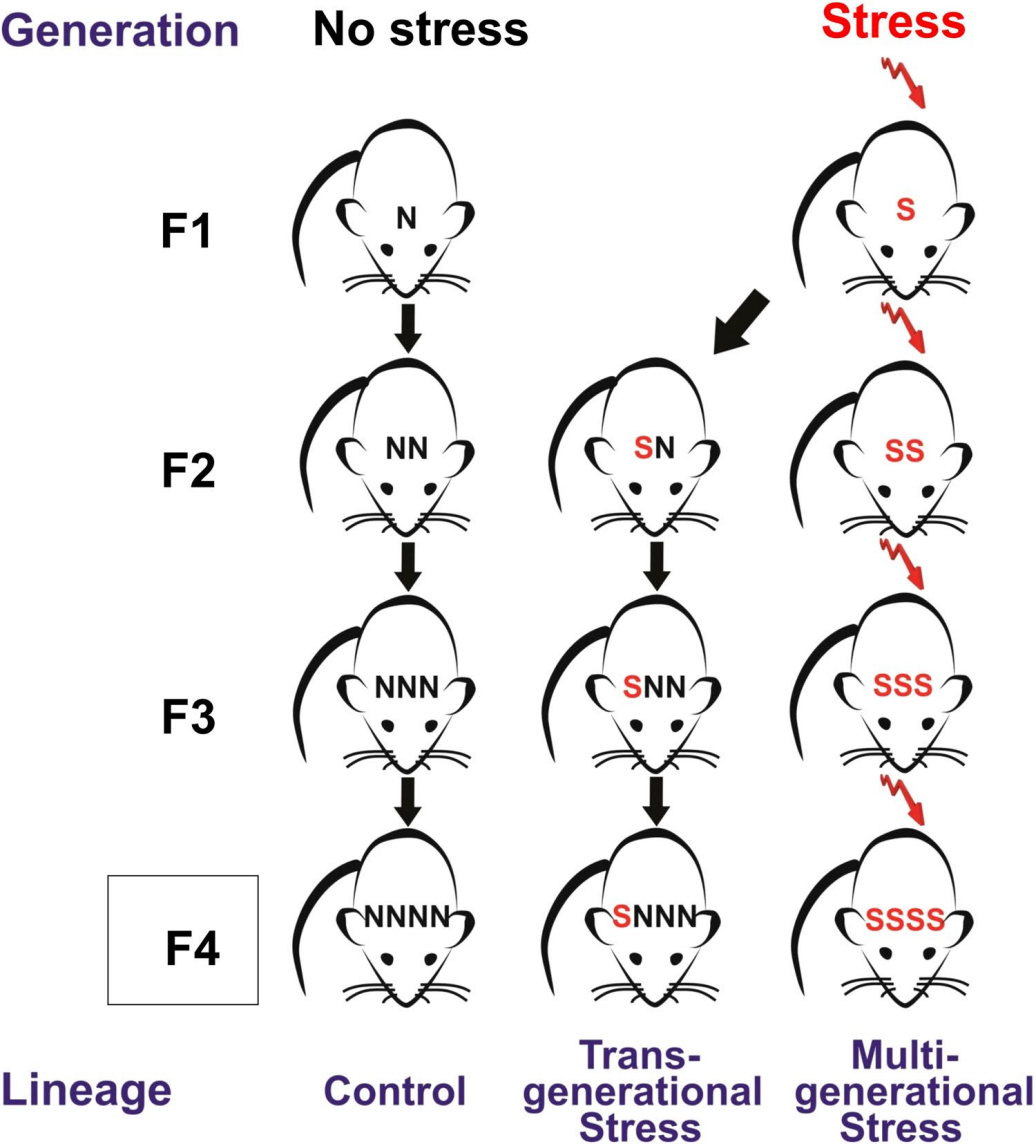
Trans- and multigenerational stress lineages. Naïve dams were stressed during timed pregnancy to generate the F1 prenatally stressed offspring (F1-S). For the F4 generation, transgenerationally stressed (F4-SNNN) offspring came from a lineage where only great-great grandmothers were exposed to stress during pregnancy, while multigenerationally stressed offspring (F4-SSSS) came from a lineage where 4 consecutive generations of pregnant mothers were subjected to stress. Housing, handling, testing and tissue sampling conditions were harmonized across generations. Each generation was outcrossed, and family trees were closely monitored by the JAX Colony Management System (JCMS).

Under standardized conditions, two different lineages of timed pregnant rats were bred (see Fig. 7), prenatally stressed (S) and non-stressed (N) conditions. In the stress condition, female rats were stressed on gestational days 12–18 by a semi-random daily sequence of 5 min swim and 20 min restraint in a Plexiglas cylinder to generate F1-S offspring. In the non-stress control condition, dams remained unstressed (N) to generate F1-N offspring. A transgenerationally stressed lineage was generated by stressing only dams of the parental F0 generation, while F1 daughters (F1-S), F2 granddaughters (F2-SN) and F3 great-granddaughters (F3-SNN) were not stressed during pregnancy. In addition, stressing dams of each consecutive (F1-F3) generation during pregnancy generated a multigenerationally stressed lineage (F1-S; F2- SS; F3-SSS and F4-SSSS; see Fig. 7). A lineage of yoked controls was bred with each generation (non-stress pregnant F0, F1-N, F2-NN, F3-NNN). Each generation F0-F4 was outcrossed avoiding inbreeding by at least four generations. Distinct lineages were monitored through the JAX Colony Management System (JCMS; Jackson Laboratory, Bar Harbour, ME, USA). A maximum of three offspring per litter of each sex were randomly selected to be included in the experiments. Each experimental group included offspring from at least 3–4 different litters. Bystander effects of stress were avoided by using designated testing and housing spaces. All housing, handling, testing and tissue sampling conditions were harmonized across generations.

By including the F1 generation with prenatal stress (F1-S), the F3 generation with transgenerational stress (F3-SNN) and multigenerational stress (F3-SSS), and the F4 generation with transgenerational stress (F4-SNNN) and multigenerational stress (F4-SSSS), the present study differentiates the direct impact of stress from truly heritable epigenetic phenotypes^16^ (see Fig. 1). The experiments included both male and female F1 animals [males: n = 20 (F1C = 10, F1-S = 8); females: n = 17: (F1-C = 9, F1-S = 8], F3 animals [males: n = 56 (F4-NNNN = 21, F4-SNNN = 18, F4-SSSS = 17); females: n = 60 (F4-NNNN = 29, F4-SNNN = 15, F4-SSSS = 16)] and F4 animals [males: n = 58 (F4-NNNN = 23, F4-SNNN = 11, F4-SSSS = 24); females: n = 66 (F4-NNNN = 24, F4-SNNN = 18, F4-SSSS = 24)]. All groups were tested for exploratory activity and anxiety-like behaviours at P90. F4 animals (SSSS and SNNN) only were also tested for long term anxiety-like behaviours at six months of age because the largest effects of ancestral stress were observed in this generation [male n = 21 (F4-NNNN = 11, F4-SSSS = 10); female n = 24 (F4-NNNN = 13, F4-SSSS = 11]. Since the multigenerational stress (F4-SSSS) induced more profound effects on anxiety-like behaviours than transgenerationally stressed offspring further neuromorphological [male n = 12 (F4-NNNN = 6, F4-SSSS = 6); female n = 12 (F4-NNNN = 6, F4-SSSS = 6)] and epigenetic [male n = 6 (F4-NNNN = 3, F4-SSSS = 3); female n = 6 (F4-NNNN = 3, F4-SSSS = 3)] analyses focused on the F4 generation.

### Prenatal Stress

Pregnant dams were stressed daily by forced swimming in warm water (22 °C) for 5 min and 20 min restraint in a Plexiglas cylinder daily on gestational days 12–18^20,21,35,36^. Stressors were administered each day in semi-random order either in the morning (9:00) or afternoon (15:00) hours.

### Behavioural Testing Open Field

Exploratory activity and anxiety-like behaviours were recorded by open field locomotor activity, a standard measurement of emotional states in rats^66^. Briefly, animals were placed individually into AccuScan activity monitoring Plexiglas boxes (length 42 cm, width 42 cm, height 30 cm) and monitored for 10 min using VersaDatTM software (AccuScan Instruments Inc., OH, USA). Total distance traveled (cm) per 10 minutes was used to measure overall activity, and the total time (sec) spent in margins was used to indicate anxiety-like behaviours.

### Elevated Plus Maze

Anxiety-like behaviour was assessed using the elevated plus maze. The ‘+’ shaped maze consisted of two open and two closed arms (each 40 cm long and 10 cm wide) and was elevated 90 cm above the ground. The open arms had no side or end walls, and the closed arms had side and end walls (40 cm high). Briefly, rats were placed individually in the central square (10 cm × 10 cm) facing either closed arm and were allowed to explore the apparatus for 5 min while being video-recorded. Following each test, the apparatus was thoroughly cleaned with 10% clinicide (Vetoquinol, Lavaltrie, QC, Canada) to eliminate the odor trace. Anxiety-related behaviour in terms of time spent risk assessing was evaluated by an experimenter blind to the experimental conditions^67^.

### Blood Collection and Analysis

Blood samples were obtained three days prior behavioural testing. On average 0.6 ml of blood was collected from the tail vein between 8:00 and 10:00 AM under 4% isoflurane anesthesia^35^. The blood was transferred to centrifuge tubes and plasma was obtained by centrifugation at 5,000 rpm for 10 minutes. The samples were stored at −86 °C. Plasma corticosterone (CORT) levels were determined by enzyme-linked immunosorbent assay (ELISA) using commercial kits (Cayman Chemical, Ann Arbor, MI, USA).

### Histological Processing for Golgi-Cox Staining

Following behavioural testing, animals were treated with an overdose of pentobarbital (Euthansol 100 mg/kg; CDMV Inc., Québec, Canada) and intracardially perfused with 0.9% saline. Brains were removed and preserved in Golgi-Cox solution for 14 days then placed in 30% sucrose for 28 days. Brains were sectioned on a vibratome (Leica, Buffalo Grove, IL, USA) at 200 µm, and slices were mounted on gelatin-coated slides. Sections underwent a Golgi-Cox staining protocol 39 for visualization of distal dendrites. Only dendritic segments that met the criteria of being thoroughly stained and without overlap with another dendrite or blood vessel were included in the examination^40^.

**Figure 8:**
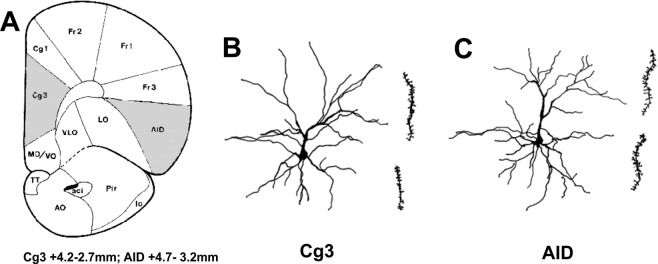
Morphometry of Golgi-stained pyramidal neurons in the medial prefrontal cortex (Cg3) and the orbital frontal cortex (AID). (**A**) Schematic diagram of a coronal section illustrating the location of Cg3 and AID (shaded areas). (**B**) Representative Cg3 pyramidal neuron showing apical and basilar dendrites and dendritic segment with spines. (**C**) Representative AID neuron showing basilar dendrites and dendritic segment with spines.

Pyramidal cells from cortical layer III, of the orbital frontal cortex (OFC/AID) and medial prefrontal cortex (mPFC/Cg3) were analyzed (Fig. 8). Individual neurons were traced from the Golgi-Cox stained brains, using a camera lucida mounted on a microscope. A total of 10 cells (5 per hemisphere) were traced at 200× magnification per animal. Neuromorphological measurements obtained from the AID and Cg3 included apical and basilar Sholl analysis (an estimate of dendritic length derived from dendritic branches that intersect concentric circles spaced 25 µm apart), apical and basilar dendritic branch order (an estimation of dendritic complexity based on the number of branch bifurcations) and spine density (the number of spine protrusions on a 40 µm-segment of dendrite traced at 1000× magnification^21,68^.

### miRNA and mRNA Deep Sequencing

Following behavioural testing, the subgroup of animals (n = 3) was treated with an overdose of pentobarbital (Euthansol 100 mg/kg; CDMV Inc., Québec, Canada), and once the vital signs were discontinued animals were decapitated. The brains were rapidly removed, dissected and flash-frozen (−80 °C) for miRNA and mRNA analysis. The TRI Reagent Solution was used to extract total RNA from the frontal cortices (Invitrogen, Carlsbad, CA, USA).

MiRNA expression analysis was performed using a Illumina GAIIx genomic analyzer (Illumina, CA, USA). Briefly, using default setting the base calling and demultiplexing was completed by the CASAVA 1.8.1 software pipeline (Illumina, CA, USA). To examine short read quality FastQC software was used. Adapters were trimmed using Cutadapt software (https://cutadapt.readthedocs.org/)^69^. Another FastQC quality check was performed after trimming. MicroRazerS version 1.0)^70^ short read aligner was used to perform miRNA mapping. Reads mapping to mature miRNAs were counted using an ad hoc bash script. Potential targets of selected miRNA of interest were predicted using the 3′ UTR available for Rat rn5 (UCSC) genome. An algorithm (miRanda v.3.3a; Computational Biology Center of Memorial Sloan-Kettering Cancer Center, NY, USA) was used for miRNA target prediction.

mRNA analysis was also performed by a Illumina GAIIx genomic analyzer (Illumina 462 Inc., San Diego, CA, USA), using multiplex. Every library was sequenced across 3 separate lanes. Base calling and demultiplexing was performed by Illumina CASAVA 1.8.1 with default settings using Rat - Rnor 5.0 (Ensembl) as reference, and sequence and annotation information were downloaded from iGENOME (Illumina). Raw count data were uploaded into R, initial data exploration and outlier detection were performed using arrayQualityMetrics and DESeq2 bioconductor packages. First raw counts underwent normalization and variance stabilization procedure as described in DESeq2 manual. Hierarchical clustering of transcriptional profiles based on top 100 most variable genes, pre-selected from the subset of highly expressed genes (higher than the median expression). Clustering was performed using the heatmap.2 function from the gplots package with default clustering algorithm. Gene expression values were displayed as heatmaps. In addition to hierarchical clustering, similarity between samples were visualized as PCA plots built using plot PCA function implemented in DESeq2. Outlier detection and transcriptional profile quality control was performed using array Quality Metrics package.

### Statistical Analysis

Statistical analysis was performed using SPSS 20 for Windows 11.5.0 (IBM Corporation, Armonk, NY, USA). Three-way ANOVA with sex, stress, and generation as factors was run for behavioural tasks at P90. Similarly, three-way ANOVA with sex, stress and hemisphere as factors was run for neuromorphology of the orbital frontal cortex (OFC; AID) and the medial prefrontal cortex (Cg). A two-way ANOVA was completed for open field activity at P180 and elevated plus maze task at P180. Tukey’s test was used for all behavioural and neuromorphological posthoc analyses when possible. Otherwise, independent sample t-test was run. For miRNA and mRNA analysis, raw count data was first normalized and regularized with log transformation using statistical routines implemented in the DESeq2 bioconductor package^69^ as described in the DESeq2 user manual. Default settings were used to perform normalization and statistical analyses. Pairwise comparisons between experimental groups (stress and non-stress) were performed using DESeq2. To be considered differently expressed, miRNA’s and mRNA’s with a false discovery rate adjusted p-values < 0.1 were used.

All results are shown as the means ± standard error of the mean (±SEM).

## Supplementary information

Supplementary Table 1
