# Supplementary material for: Ancestral Stress Alters Lifetime Mental Health Trajectories and Cortical Neuromorphology via Epigenetic Regulation
Source: Sci Rep. 2019 Apr 23;9:6389. doi: 10.1038/s41598-019-42691-z (PMC6476877; doi:10.1038/s41598-019-42691-z)
Supplement: Supplementary file 1 — Supplementary Table 1 [file 41598_2019_42691_MOESM1_ESM.docx]

**Ancestral Stress Alters Lifetime Mental Health Trajectories and Cortical**

**Neuromorphology via Epigenetic Regulation**

*Mirela Ambeskovic*¹, Olena Babenko¹, Yaroslav Ilnytskyy*²*, Igor Kovalchuk*²*, Bryan Kolb¹,*

*Gerlinde A.S. Metz¹*

| **miRNA** | **Pathway** | **Target Genes** |
| --- | --- | --- |
| Mir 26 | postsynapse3 | Targets: ABL2, ABLIM1, ACTN2, ADAM10, ADD3, ANK2, ANK3, ANKS1B, APP, ARF1, ARFGEF2, ARHGAP32, ARHGAP44, ARHGEF2, ASAP1, ATAD1, ATP1A2, BCAS1, BCR, BMPR2, BSN, CACNA1C, CACNG8, CAMK2A, CAMK2G, CANX, CBLN1, CDK5R1, CHRM3, CHRNA5, CHRNB1, CHRNB2, CNKSR2, CPEB1, CPEB4, CTNND2, CTTNBP2, DAB1, DBNL, DCLK1, DGKI, DLG1, DLG2, DLG4, DMD, DNAJC6, DNM3, DTNBP1, EPB41L1, EPB41L3, EPHA4, EPHA7, EPS8, ERBB2, FBXO45, FXR1, GABRA1, GABRA2, GABRA4, GABRB2, GABRB3, GABRG1, GABRG2, GLRA1, GLRA2, GLRA3, GOPC, GPHN, GRIA2, GRIA3, GRIA4, GRIK2, GRIN2A, GRIN3A, GRM1, GSK3B, HOMER1, IGF2BP1, IL1R1, IQSEC3, ITGA8, ITPR1, KCNC2, KCND2, KCNJ2, KCTD16, LIN7A, LPAR1, LRP8, LRRC7, LRRTM1, LRRTM2, LRRTM4, MAGEE1, MAP1A, MAP1B, MAP2, MAP3K7, MIB1, MINK1, MPDZ, MYH10, NEFH, NETO1, NETO2, NLGN1, NLGN2, NLGN4X, NOS1, NTRK2, OPHN1, PAK1, PALMD, PDE4B, PDLIM5, PDPK1, PJA2, PLCB4, PPP1R9A, PSD3, PTCH1, PTEN, PTPRO, RIMS1, SH2D5, SHANK2, SHC4, SLC1A3, SORCS3, SOS1, SPTBN1, STRN, SYNE1, TENM2, UTRN, VPS35 |

**Supplementary Table 1.** Table illustrating miR-26 targets (MAP1A and NTRK2) via postsynapse3 pathway as per GO Cellular Component database.
